# Extra Supplement.—The Nursing Mirror

**Published:** 1893-10-07

**Authors:** 


### "The Hospital" Nursing Mirror.

Being the Extra Nursing Supplement of "The Hospital" Newspaper.

[Contributions for this Supplement should be addressed to the Editor, The Hospital, 428, Strand, London, W.C., and should have the word "Nursing" plainly written in left-hand top corner of the envelope.]

### News from the Nursing World.

### THE NURSE DOLLS.

The Hospital collection of nurse dolls has been much appreciated at Keighley, and the Secretary writes: "I am glad to say that our exhibition has been a success. . . . The children's ward in the new wing of our cottage hospital will, we hope, receive a clear £100. Your collection has been much admired. Most people have taken a great interest in it, and we are deeply grateful for your kindness in loaning us such a collection."

### DELICATE CHILDREN.

A small Home for Convalescent Children is doing good work quietly at 66, St. Peter's Road, South Croydon. Any delicate children who do not require night nursing nor medical attendance are eligible, and necessitous cases are taken free. Patients whose friends are able to pay are charged the moderate sum of 5s. 6d. weekly. The rules, which are very simple ones, and other particulars, can be obtained on application to "Sister," at above address.

### WHAT BECOMES OF OLD LINEN?

Old boots, old hats, and old clothes generally are easily disposed of. There is always somebody who is a little, or a great deal worse off than ourselves, who is glad to receive any or all of these cast-off garments. But what becomes of all the linen which is annually condemned by notable housekeepers? We have been asking the district nurses, and they say they only wish they knew! But, anyway, they don't get it themselves; they wish they did. Lint, beautiful fair rolls of spotless lint, seems indigenous to the soil in hospitals, but the district nurse has to make the best of a niggardly portion of clean, old rag. "If only there were more of t," she often sighs as she packs up her basket; "and I know there are presses full of worn linen in lots of large houses, which only want 'looking through.'" Well, the nurses' "season" has begun, it is a time of special sickness and distress, and surely the ministrations of these invaluable visitors should not be hampered by lack of materials. As regards "sorting over" or "looking through" piles of well-worn sheets, table-linen, and underclothing, surely that need not be a hindrance? We shrewdly suspect that, however large the bale sent, a good use would be found for every scrap of the contents by the superintendent of any district nursing association—such, for instance, as the Rural Branch of the Queen's Jubilee Institute.

### NURSES' CO-OPERATION.

The characteristics of the original Nurses' Co-operation are mainly these two: It is managed by a committee, and the members receive their own earnings, less a small percentage for working expenses. The movement has been widely supported. It is not only the first scheme of the kind to take root in England, but it may be accounted as an exceedingly successful one, both as regards the nurses and also their employers. Institutions which professedly follow on the lines of the original Nurses' Co-operation appear to differ from it in very essential points, such as the absence of a published annual report showing expenditure and profit, and the exact rate of the percentage demanded from those who join. It is certainly most desirable that qualified and certificated nurses desirous of becoming members of any existing association, should thoroughly understand, before pledging themselves, what obligations are thereby incurred, and also the exact nature of the agreement which they enter into.

### RURAL REQUIREMENTS.

"We want you to give our villagers a few simple talks on home nursing," wrote the Squire's wife to a lady specially trained to give practical instruction in plain language to rural audiences. The outlines of a scheme were sketched out and forwarded promptly for approval. It was drawn on distinctly common-sense lines, and avoided all poaching on the doctor's province and other such popular incongruities. By return of post the experienced teacher received back from the inexperienced country dame the copy of her syllabus, with a note, "kindly insert following points, your ideas of the cottagers requirements are certainly not up to date!" The trained nurse regarded with fear and dismay the additions demanded, for, amongst others, she read: "Special treatment of pneumonia, bronchitis, heart disease, and pleurisy. Treatment by subcutaneous and other injections, special (hospital) appliances and the treatment and diagnosis of nervous diseases." "Simple talks on home nursing" seem likely to be crowded out by "medical mixtures up to date," concluded the lecturer, as she wrote a courteously firm refusal to have any share whatever in the propagation of a medley containing such dangerous ingredients.

### WORKHOUSE NURSES AND CERTIFICATES.

The institution of probationers in the infirmaries of workhouses is a decided step forward, and cannot fail to benefit both the overworked nurses and the patients. A provincial union is now about to grant certificates to probationers after two years' training, but some of our readers will be shocked to learn that no examination precedes the bestowal of the certificate.

### MEDICAL WOMEN.

A large company, chiefly composed of past and present students and their friends, assembled at the London School of Medicine for Women on October 2nd. With exemplary punctuality the proceedings began at half-past three, when Mrs. Garrett Anderson, dean of the school, took the chair, and Miss Helen Webb, M.B., physician to out-patients at the New Hospital for Women, and lecturer at the Clapham Maternity Hospital, gave the opening address. She spoke in a most practical manner to the young ladies who were about to take up the study of medicine, and bid them let method and neatness characterise their work, their persons, and their surroundings. She also, in advising proper care of their own health, bid them aim at an ideal state in which the medical profession would be an unnecessary one. The conclusion of Miss Webb's very eloquent speech referred to the work for which women doctors were specially required. The School of Medicine for Women is certainly a most successful modern development.

### ANOTHER QUEEN'S NURSE.

A well-attended meeting was recently held at Banchory, N.B., and it was unanimously agreed to form a local committee and establish a Queen's Nurse in the town. Many subscriptions were volunteered, so there appears every likelihood of the adjoining districts as well as Banchory itself each possessing eventually a permanent Queen's Nurse.

### NURSING AT WASHINGTON.

Five years ago the Garfield Memorial Hospital at Washington established a training school for nurses. The general standard of the institution was so much improved by the change in the nursing system that other hospitals adopted the same plan, and the city now possesses four regular schools. The Garfield Memorial Hospital consists of four connected buildings located on a beautiful site overlooking the city and the Potomac. Washington also possesses separate hospitals for women, children, coloured people, Catholics, an emergency, and a homœopathic establishment. The Garfield, which is a general hospital and unsectarian, is open to all without distinction of race or colour. Nurses are better paid than they used to be, and are required to be fully qualified.

### QUALIFIED FOR INDIA.

The valedictory meeting held at St. Martin's Town Hall last week was one of unusual interest, on account of the number of missionaries on the eve of departure for the great Indian Empire. The Zenana Bible and Medical Mission should be cordially commended and supported, because it sends out only fully qualified medical women. The necessity for this precaution can hardly be overestimated, and should be constantly insisted upon. All Hospital readers are aware of the disasters resulting from unqualified assistants, as well as untrained monthly nurses, practising in isolated country parishes. Yet the risks are as nothing when compared with the proceedings of half-trained female medical students or uncertificated midwives pursuing their callings in remote Indian villages. They are quite out of reach of competent assistance, and they are also safe from the professional supervision which in thickly populated countries such as England would inevitably bring public discredit on their lamentable ignorance. Societies such as the Zenana Medical Mission will do much to raise the standard of medical and nursing work in India, and we may reasonably hope that a shortened medical course, or a limited allowance of training, will eventually disqualify candidates for Indian appointments, just as they would for similar and even less responsible posts in Great Britain.

### PRIVATE NURSES IN INDIA.

English trained nurses, we are often told, are appreciated and required in India, in fact, people go so far sometimes as to say, "urgently needed." Both facts may be literally true, but there is another side of the question which should receive equal publicity. In order to enable them to live during the months when there is no demand for their services, nurses are obliged to ask such high fees that only a limited number of families can afford the luxury of employing them. Hence private hospitals, with inclusive and reasonable fees, get well supported, whilst the unfortunate private nurse is often for months without work, in a country where the necessaries of life are most costly. Therefore all who aspire to try nursing "on their own account" in India should realise that work is intermittent, and although high fees may be the rule, sick persons able to pay them are quite the exception.

### INACCURACIES.

Our correspondents seem to have taken heed to our warning that all unsigned communications, of whatever kind, must be content to find a home in the waste paper basket. At any rate, full names and complete addresses are now generally appended. But there are still points which need attention, notably such as the formation of figures. What is the use of an address in which the numbers are so imperfectly formed that they baffle even the experts in the Post Office? Nurses should take special care to give a full and correct direction, otherwise they cause delay and give unnecessary trouble. "Nurse Emily" requested some information recently, which was obtained for her and promptly forwarded, but through some inaccuracy the letters failed to reach their destination, and were returned on our hands as "incorrectly addressed," which is discouraging to those kindly desirous of advising her.

### SHORT ITEMS.

The Littleborough cyclists held a fancy dress parade the other day, which resulted in £20 being raised for the District Nurse Fund. The entertainment was excellently managed, and it is to be hoped that a similar fête will be organised annually. The nurse's work seems to be highly appreciated at Littleborough, and in such a large district there must be plenty of occupation for a second worker.—A new convalescent home was recently opened at Hexham, in connection with the Newcastle Cathedral Nurse and Loan Society.—Mrs. Bedford Fenwick, who has been a contributor from the first, has now become the editor of the *Nursing Record*.—All work intended for our Christmas Competition should be sent to The Hospital office during the first week in December; particulars as to the kind of garments, &c., appeared in last week's journal.—Miss Rooke, who lectured in Leicestershire under the County Council, has been engaged to give a course on Nursing for the Lincolnshire County Council.—We hear that the land on which Trimmer's Cottage Hospital is to be erected was given by the sons of the late Mr. George Trimmer, and not by Mr. Knight, of Farnham. Fifteen thousand pounds was the sum dedicated by Mr. G. Trimmer for this admirable purpose.—A fire broke out at the Small-pox Hospital at Bradford on 2nd inst. It was impossible to save the building, but the patients, seventy in number, were all rescued, thanks to the prompt action of Miss Mellor and her nursing staff. The patients were wrapped in blankets and placed on mattresses in an adjoining field. Within an hour of the outbreak, the doctors and members of the Sanitary Committee had all the sick safely housed in the Fever Hospital three miles away.

### On the Nursing of Diseases of the Nervous System.

### I.—INTRODUCTORY.

A short consideration of the structure and functions of the nervous system must precede the account of the diseases of that system.

Nervous tissue consists of nerve fibres and nerve cells; the former starting from the latter, and dependent on them for their life. Along nerve fibres nervous impulses are sent; thus we may compare the fibres to telegraph wires. Nerve cells originate nervous impulses, or receive them and send them out again. These may similarly be compared to telegraph offices.

The nerve cells are found massed together in the grey matter of the brain and spinal cord; in other places they are grouped together forming ganglia. Nerve fibres are contained in the white matter of the brain and spinal cord, and make up the nerves.

The picture and diagram of the brain in Huxley's "Elementary Physiology" give clearly the relative position of its different parts.

The cerebrum consists of two hemispheres, separated by a deep fissure. The surface of each cerebral hemisphere is composed of grey matter, which is divided by fissures or depressions into convolutions. Inside each hemisphere is a cavity or ventricle containing some clear fluid. These two ventricles communicate with a medianly-placed ventricle in the next part of the brain, and this third ventricle communicates by a narrow passage under the corpora quadrigemina with a fourth ventricle, and from the hind end of this opens a small canal, which continues through the centre of the spinal cord.

The cerebrum is the seat of origination of voluntary movements and intellectual processes. It rules over other parts of the brain which are engaged in arranging and sending out nervous impulses.

The cerebellum consists of two lateral hemispheres connected by a median portion. It lies behind and below the corpora quadrigemina, to which it is attached by two peduncles. Two peduncles also pass from it to the Pons Varolii, and two inferior peduncles from it to the medulla oblongata or bulb.

From the brain come off twelve pairs of cranial nerves. Thus the first pair are nerves of smell; second pair, nerves of sight; third, fourth, and sixth pair supply muscles of the eyeball; fifth pair are sensory nerves of the face and head, and also supply the muscles of mastication; seventh pair supply the muscles of the face and scalp; eighth pair are nerves of hearing; the ninth nerves of taste; the tenth are the vagi that supply the larynx, lungs, heart, and stomach; the eleventh pair supply some muscles of the neck; and the twelfth the muscles of the tongue.

The spinal cord when cut across is seen to consist of grey matter, more or less in the form of an H, with two horns directed forwards, the anterior cornua; and two directed backwards, the posterior cornua. Around the grey matter is white matter, divided by anterior and posterior fissures into two halves. In connection with the cornua are the nerve roots, which are thus anterior and posterior, and these unite to form a nerve trunk. There are thirty-one pairs of nerves coming off from the spinal cord. Nervous impulses pass along the anterior nerve roots from the central nervous system, then along the nerve trunks, and so to the muscles and other parts of the body supplied by the nerves. Such nervous impulses are *efferent*, as they pass out of the central nervous system. Nervous impulses, passing towards the central nervous system, *i.e.*, *afferent* nervous impulses, reach it along the posterior roots. There are thus nervous impulses passing in two directions along different paths; the paths or tracts conveying impulses from the central nervous system to distal parts are motor tracts, the tracts conveying impulses in the opposite direction are sensory tracts. The motor tracts begin in the cortex or superficial grey matter of the cerebral hemispheres, pass through other parts of the brain, and at the lower border of the medulla cross to the opposite side of the spinal cord, and then out at the anterior nerve roots. Thus the right half of the brain governs the left half of the body, and the left half of the brain the right half of the body. Similarly the sensory impulses, on their way from distal parts to the brain, cross to the opposite side in some part of their course.

In disease we shall find that either the motor or sensory tracts are mainly affected. The disease may begin in the brain, spinal cord, or peripheral nerves. Both the brain and spinal cord are invested in coverings, or membranes (meninges), with in the bony cavities which contain them, and that membrane next the brain or spinal cord carries the blood vessels to those structures. These membranes may also be the seat of disease. When the perverted nervous function is dependent upon a diseased condition in any part of the nervous system, such a condition being recognisable on examination, it is said to be due to structural disease. A disease is said to be functional or a "neurosis" when there is no organic change in any part of the nervous system, but nervous symptoms exist. The distinction between these two forms of disease is very important, and to arrive at a diagnosis of functional disease, most careful exclusion of organic disease must be made, and, of course, to do this there must be an accurate knowledge of the groups of symptoms that occur in different forms of disease. Hysteria gives rise to the greatest number of difficulties, and it may simulate many organic diseases. A nurse may render the greatest assistance in such cases by careful observation and by firm and judicious treatment of the patient. But we shall at the present time consider organic disease of the nervous system, beginning with diseases of the brain.

Before considering the symptoms of these diseases, it will be well to mention the mechanisms by which damage to the brain is produced. First, there may be destruction of the cerebral tissues, or there may be pressure not causing actual destruction, but rendering the nerve fibres incapable of carrying nervous impulses. Pressure suddenly produced, as by an effusion of blood, gives rise to more marked symptoms than pressure gradually produced, as by a slowly-growing tumour. Secondly, inflammation may occur, leading to degeneration of the nervous tissues. Thirdly, the blood supply to parts of the brain may be completely stopped, causing death of the nervous tissues in the area supplied by the vessels.

Damage to the nervous tissues is evidenced either as irritation or loss of function. In irritation nerve fibres are stimulated excessively, for example, in convulsions. In loss of function there is loss of movement or loss of sensation. The onset of symptoms may be sudden, acute, or chronic. A sudden onset would mean that the symptoms developed either in a few minutes, or, at any rate, not longer than in a few hours; the cause in such cases would generally be rupture or blocking of a blood vessel. In an acute onset the symptoms would come on in time varying from a day, to two or three weeks; then probably there is some inflammatory trouble. If the symptoms develop more slowly still, coming on gradually in the course of a month or more, then probably the damage is due either to some growth in the brain, chronic inflammation, or degenerative changes.

### Nursing in Paris.

(By a Wandering Nurse.)

### III.—A VISIT TO THE CHILDREN.

Perhaps it was only natural that after spending some time amongst the adult patients at the Hotel Dieu, I should begin to think of Children's Hospitals by way of a variety.

Of course there must be several of these in such a city as Paris, but the question was firstly, how to find them, and secondly, how to get permission to enter.

It was not difficult to obtain the addresses, and I copied down three from the hotel directory; then I went and had a talk with a "commissionaire," an excellent man, who had already given me valuable hints as to the best way of getting to places.

Eventually I decided, at his suggestion, to take a certain omnibus which, he pointed out, would put me down at the "Hôpital des Enfants," situated near the Rue de Sèvres.

The necessity for some permission or introduction still remained to be met, and both English and American acquaintances had assured me that I could not possibly get in, unless armed with something of this kind.

I was busy sight-seeing, "making the most of" my time, till the end of the week, but on Sunday, over my solitary lunch, I bethought me again of the unvisited Children's Hospital. "I declare I will try and get in to-day," I said to myself; "perhaps there is a visiting hour on Sundays."

The omnibus recommended by the friendly commissionaire conveyed me to the gates, set in a long white wall, where already a group of people were assembling. I asked a stately gendarme, in the best French I could muster, at what hour the visitors were admitted, and his reply showed that I had but a quarter of an hour to wait. Women were selling flowers, cakes, sweetmeats, and biscuits along the edge of the pavement. Finding it was permitted to take in these to the patients, I bought a few packets of the least indigestible-looking and also a lovely spray of roses. During the first purchase an amusing incident occurred. I had spent a franc with one picturesque-looking old woman, and walked a few yards away from her, when it occurred to me that my two packets would not go far, and I might as well invest in some others. In all innocence I accosted a different woman, who was as picturesque and smiling as the first one, and soon got what I wanted from her. However, the other was deeply affronted by what she evidently considered my preference for the appearance of her neighbour's basket of wares, and she uttered so many biting sarcasms that I hastily asked her to oblige me with a further consignment of her biscuits, just to show that I liked all kinds equally. She was somewhat appeased, but continued to argue further with her neighbour, whilst I passed on, thinking that possibly the latter was quite equal to defending her own action without any backing up from her customers.

The clock was striking by this time, and the people passed in through the side gate, at which stood a neat-looking nurse, with a pretty French cap poised on her shining hair.

Each person on entering seemed to address her, and I therefore did the same. I said that I was a stranger, knew none of the patients, but should be glad to go and see the children. She consented at once, and I found that this was the only day and hour in the week when this freedom was permitted. The long, straight pathway was bordered by detached houses, which had plenty of open land around and behind each block of buildings.

A brisk Parisian overtook me, talking rapidly about her sister's "little one," evidently inviting my sympathy. So I asked her to explain, and then she described, with much vivacity, the arrival on the previous day of a letter, telling of the child's urgent illness, and begging her to visit it on this Sunday in place of the mother, who could not come.

In return for my interest, the aunt inquired kindly who I was myself going to see, and then we agreed to proceed together to the crêche where the little one lay.

It proved to be a building on the right hand side, and we entered an old-fashioned doorway, and mounted a well-worn staircase, finally reaching two rooms, each containing a number of cots.

The children were all under one year, and very puny little creatures most of them seemed. They were all beautifully clean, and so were their plain and homely apartments. Each infant wore a little coloured jacket over its night gown, and was rolled closely up in a blanket which seemed to reach from the arm-pits far below the feet.

The bottles from which they were fed were round ones, slightly flattened out, and with no tubes but only the short India-rubber nipples. The bottles were all beautifully clean and bright, the milk was accurately measured and sterilised. The feeding bottles generally used were marked off, like medicine glasses, which seemed a wise arrangement.

It is often so difficult to know exactly what amount of nourishment is taken by babies. "I've given him a bottleful" is such a misleading statement, for the vessel may still contain half the quantity when it is removed later on.

These French nurses all asserted that the quantity given at each time to a baby was satisfactorily sucked up by the end of ten minutes. Certainly those bottles which I saw taken away from the beds were quite empty, and I noticed that the removal was as systematic as the giving of them round to the poor little sick babies.

### An Isolated Small-pox Case.

(By One of the Nurses.)

"Well, nurse, what do you think of the place?" said a pleasant voice behind me, and turning round I saw a young man standing just inside the door, whom I rightly concluded was the doctor.

"It's the funniest place I was ever in," I replied, laughing. The blazing firelight showed a little whitewashed room, bare of furniture, save for a small bed and one wooden chair, and on the bed lay a man, his head swollen and disfigured, his hands wandering restlessly after the shadows on the wall. Every now and then he raised himself on his elbow and peered about him in the way trained nurses recognize so easily as a sign of delirium.

"Well, ask for anything you want, and see that you get it," continued the doctor. "Do you think you can manage alone to-night? To-morrow I will get another nurse to help you, but I have found it impossible to-day."

"I can manage very well," I replied, not telling him that I had already asked if I could make any one hear if help was needed in the night. I had been quietly told "no one would hear, nor would they come if they did." After giving his directions, the doctor said good night, promising to come early in the morning.

Have you ever seen a person suffering from confluent small-pox? it is not a pleasant sight, and I must own to a slight feeling of fear as I heard the doctor's steps die away, and I realized that I was now alone in a small cottage, far from all other houses, with a tramp already delirious and likely to get worse. The poor fellow had been taken ill in a workhouse, and had been removed to this empty cottage for the purpose of isolating the case, and truly the completeness of the isolation forced itself at that moment most unpleasantly on my mind.

As the night advanced, the restlessness of my patient increased, and at last I found it impossible to keep him in bed, for his strength was greater than mine, so I just locked the door and stood in front of the window to keep him from jumping out.

For a time he rambled about the room, muttering to himself, or shouting hoarsely at the imaginary enemies surrounding him. Towards morning I coaxed him to get into bed, and then he fell into a doze and I felt it was safe to leave him for a minute, and went into the next room to make a cup of tea. Feeling hungry I cooked an egg, but found some difficulty in eating it, as a two pronged iron fork was the only implement I could find.

During the night a cat looked in, and thinking perhaps I was lonely, or that it might find the room more comfortable than the rain outside, he went and fetched in his family. On my return from the other room by-and-by, I found him sitting by the side of his black and white wife in front of the fire, whilst their only child was curled up like a very damp and muddy ball on my bed. They all stayed with me during the rest of my residence at H——, and I cordially reciprocated their kind friendship. The next night, to my delight, another nurse arrived, and then we managed quite comfortably. We had a sort of Box and Cox life with the bed, one sleeping in it by day and the other by night. The life was a rough one, but as our patient went on well, we were relieved from anxiety on his behalf, and managed to extract a good deal of fun out of the shifts we were put to. For instance, the draughts were so great, that on a windy night I have often gone to sleep, laughing to think of the look of horror on my sisters' faces could they have seen me with my head under my umbrella, and my tooth-brush stuck into the latch of the door to prevent its flying open.

Everyone was kind to us, doctors, rector, and Guardians, the wife of the rector keeping us well supplied with books, and sending many little dainties, so on the whole we were very happy.

A good deal has been said and written against nurses being compelled to do menial work in the course of their training, but I was glad of all the practical experiences I had had, as owing to our state of isolation we had to clean our grates and sweep the rooms ourselves, or leave them dirty, and the alternative seemed to us quite impossible.

Day by day our patient improved, till he was able to leave his room and creep out to sit in the sunshine, and watch the gambols of a highly original donkey, who used to rush about the meadow like a mad thing. His great delight was to get hold of the mop, then he would tear round, shaking it in his teeth like a dog with a rat. He often used to chase me if I ventured too far into the field, I think he wanted to get at my cap. One very wet day I heard a loud clattering, and down the path came the doctor at full speed, his dripping umbrella waving aloft, and the donkey after him, snorting with delight.

I am glad to say that at the end of a month I was able to leave my patient so far recovered as to do full justice to his lunch, which I last saw him eating. It consisted of a hunch of bread and cheese, and half a pint of beer, and I returned to London very well pleased with the results of my experience of small-pox nursing and "complete isolation" of the patient.

### Appointments.

[It is requested that successful candidates will send a copy of their applications and testimonials, with date of election, to The Editor, The Lodge, Porchester Square, W.]

Worthing Infirmary. — Miss Lucy White has been appointed Matron at this infirmary. She was trained at Addenbrooke's Hospital, Cambridge, and was afterwards Assistant Nurse at the Sussex County Hospital, Brighton, then Ward Nurse at Worthing Infirmary, Head Nurse at East Sussex Hospital, Hastings, and for the last four years Miss White has held the responsible post of Matron at the Brecon County Infirmary. We wish her every success in her new post, which her previous experience specially fits her to fill.

### Nursing in Ceylon.

### THE CEYLON NURSES' ASSOCIATION.

Trained English nurses for English residents have long been desired in Ceylon, and they have now become an established fact, mainly through the instrumentality of Mrs. Pole Carew, who holds the post of hon. secretary to the association which she has succeeded in organizing. A private nurse who ventured out to Ceylon on her own account last spring, has been incessantly engaged ever since, and is about to join the association, of which we append the rules. Two other nurses are engaged, and more will be added by degrees, if the scheme prospers, as it ought. A small bungalow is being specially built for the nurses, and the English population has gladly subscribed towards paying the rent and other attendant expenses. The nurses are for the service of English people only, as these have hitherto been quite unprovided with trained nurses, whilst Government has supplied hospitals all over the island for natives.

Rules of the Ceylon Nurses' Association.

1. The nurses will be bound to the association for three years. The agreement to be signed within twenty-four hours of their landing in Colombo.
2. A nurse will receive 30 rupees a-week when at a case, and will pay one rupee a day towards her expenses when at the home.
3. In case of sickness, a nurse will be nursed at the home, and attended by the doctor free.
4. Each nurse will receive £15 towards her passage money.
5. When on duty a nurse will wear white uniform, consisting of plain white washing gowns, Eton collars, turned back cuffs, and white aprons, with bibs.
6. A nurse will be entitled to one month's holiday in each year.
7. Should a nurse wish to leave before her three years engagement has expired, she must refund the £15 given her for passage money.
8. Should a nurse wish to return to England at the end of three years she will be allowed £10 towards her passage, at the end of four years she will be allowed £15, and at the end of six years £30.
9. Should a nurse be reported by a doctor to the committee for misconduct or neglect of duty, she will be liable to dismissal.

### Nursing in South Africa.

(By a Practical Nurse.)

### PRIVATE NURSING.

The second experience of private nursing in South Africa was with a lady with pneumonia. It was not a very serious case, but rather a protracted one, as the patent had had a relapse a few days before I went to her, and I was told that the temperature having gone down satisfactorily, the doctor said poultices might be discontinued in the evening, but the patient was so comfortable that she begged to be allowed to keep them on. She slept from nine p.m. to five a.m., and awoke feeling cold and clammy. Fever returned, and systematic poulticing had to be recommenced. Before I was sent for the lady's two daughters shared the night nursing between them, and managed excellently, keeping very good diet charts, but no record of the temperature, which the doctor took night and morning when he visited the patient. The usual routine was pursued, nourishment every one and a-half or two hours, medicine every four hours, stimulant every four hours.

The poultices were very well made, though I found that several bottles of olive oil had been used in their composition. This was mixed in with the linseed, from a belief that they would thus be kept from sticking. The doctor had told the girls that they might use the poultices a second time, so they said they stirred the linseed up again in fresh boiling water and applied them. This rather reminded me of a case in a village at home, when I went to help to nurse a poor girl suffering from peritonitis. I found her lying with a large flat brown cake wrapped up in a towel applied to her abdomen: this was the poultice. It had been repeatedly heated up in the oven, and was reduced to a hard, heavy mass, which was certainly exactly opposite to the treatment desired by the doctor. However, my patient eventually made satisfactory progress, and there were only occasional fits of coughing, which did not last long. Two or three mouthfuls of hot water prescribed by the doctor proved beneficial in soothing the irritation, and hence stopped the cough.

In The Hospital for August 5th I notice Nurse Edith's questions about the Cape Mission. I hope my last letter, giving some account of this work, was of some use to her. The Cape General Mission has a branch in Johannesburg, but has no nursing staff connected with it. If Nurse Edith applied to the lady whose address I gave last week she will have learnt all particulars.

As to "private nursing on her own account," I think that a conscientious woman, with really good references and certificates, could find plenty of work to do, the demand for skilled workers being great both at the Cape and in Johannesburg. Printed testimonials are little valued here, and it is needful for a nurse to come out provided with her original certificates and with reliable testimonials of recent date. Letters from Matrons of hospitals or Superintendents of well-known institutions are, of course, especially useful both as proofs of training and also as guarantees of special fitness for the responsible work of private nursing in South Africa.

### Irish Hospitals.

(By a Trained Nurse.)

### A VISIT TO A CORK HOSPITAL.

The City and County of Cork Women and Children's Hospital, Infirmary Road, Cork, is well worth a visit. It is situated in the poorest quarter of the town, and its beds are kept constantly occupied. The wards are bright and pretty, and spotlessly clean. The lavatories and bath-rooms are fitted up with the latest improvements.

The probationers are carefully and thoroughly trained in all branches of women and children's nursing. Two years is considered the actual period of training, but at the end of that time nurses are expected to continue in the service of the hospital, either in the wards or on the private staff, for at least one year. The nurses and probationers have comfortable bed-rooms and an excellent dining-room, which also serves as a sitting-room, but among the pending alterations a pleasant, large sitting-room is being arranged for the nurses.

The hospital contains between sixty and seventy beds and cots, several of which are set aside for paying patients; the charges are very reasonable, and come within the means of many who could not afford to enter an expensive nursing home. The nursing staff numbers thirty-six, including a few private nurses. The hours on duty are from seven a.m. to eight p.m., with two or three hours off duty every day. The night nurses go on duty at eight p.m., and remain on until half past seven next morning.

The nurses present a particularly clean and fresh appearance in white dresses, whilst the probationers wear light blue. This hospital is the only one in Cork which trains its own nurses. Other hospitals are nursed by the nuns, except the South Cork Infirmary, where nurses are sent to be trained from a private nursing institution in the town; the infirmary possesses no nursing staff of its own whatever, and judging from outside appearances there exists room for considerable improvement. The high class nursing and the many recent improvements at the Women and Children's Hospital have all been brought about during the last five years by the skill and untiring energy of the present matron, Miss Baxter, who has been indefatigable in devising ways and means for collecting money in aid of this useful charity. There are still many improvements which she is anxious to accomplish, but owing to want of funds the committee do not see their way to carrying them out at present. It is really a Protestant institution, but many Roman Catholic patients are admitted, and as a general rule they prefer going there than to their own hospitals, where possibly the discipline is severer and the actual nursing inferior.

### Everybody's Opinion.

[Correspondence on all subjects is invited, but we cannot in any way be responsib e for the opinions expressed by our correspondents. No communications can be entertained if the name and address of the correspondent is not given, or unless one side of the paper only be written on.]

### DETERIORATION OF NURSES.

"A Sussex Nurse" writes: Owing to the recent correspondence in The Hospital on the "Deterioration of Nurses" I feel it my duty to write a few lines in defence of my fellow-workers. Some people tell us that girls' object in taking up this grand work is that they may be able to wear becoming uniforms, or to flirt with students, or that they may be privileged enough to seek and to find a husband among the staff. I think I am justified in saying that during my three years' experience in hospital work I have never yet come across a nurse whose ambition it was to obtain any of these advantages (?). Such people there may be, but they are few and far between, and probably generally fail in their object. I think perhaps it is a mistake for girls to take up nursing work when very young and unsophisticated, for not unfrequently, out of pure thoughtlessness and ignorance, they act unwisely, and there are always people ready to put an unkind construction on an otherwise innocent action. It is especially difficult an undertaking for a bright and lively girl, who at heart is aiming to lead a higher and nobler life, perhaps than people give her credit for. It would, perhaps, be better for her to wait before undertaking so responsible a work. As to outdoor uniform attracting attention, a woman who endeavours to attract attention at all will do it in whatever costume she may appear in. To a modest woman uniform is undeniably a protection. In any case attraction depends more on the wearer than on the apparel. A thorough nurse should be a thorough woman, and, above all, a through Christian. She will influence her fellow-workers for good more by action than words in many cases, and ought to do her patients good too, not merely look on them as "cases." To be a nurse is, I think, one of the highest careers for a woman, who should feel it a privilege to attend on the sick and dying, for His sake who said, "I was sick and ye visited me."

### EVERY-DAY DANGERS.

"Another Sufferer" writes: I have been the more interested in the notices in The Hospital, referring to a case of diphtheria at the Watford District Cottage Hospital, from having had myself an almost identical experience when acting as Nurse-Matron to a Cottage Hospital not many miles from London. There were five or six patients already in the institution, when a child suffering from membranous croup was brought for the immediate operation of tracheotomy, and my only assistant was a general servant. No further help was sent me until sixty-three hours after the operation, though the case was complicated and very serious, and could not be left for a single instant. Owing to the extreme kindness of the "assistant" of the medical man in charge of the case, who volunteered to keep guard for a couple of hours during each night, I was able to get four hours' rest out of these sixty-three, but nevertheless, was completely exhausted before help arrived.

After this I was relieved of the night duty, but still had to give constant attendance by day, until I myself fell a victim to the infection; the result most probably, as Miss Hillyer remarks, of being at the time "mentally and bodily over-taxed." The life of the little patient was saved, and every kindness was shown to me during my illness by the Hospital Committee. Probably in no other circumstances does both day and night duty fall to the same extent upon a nurse as in a cottage hospital. A serious case may require to be watched the whole night through, or to be visited at intervals of three or four hours for many nights in succession, and such service is always cheerfully rendered by superintendents of small hospitals. When additional help is really needed, an extra nurse should be at once engaged; the risk of "every-day dangers" cannot be avoided, but need not be unnecessarily courted and met half-way.

### THE WORLD OF NURSES.

"An Unprofessional Correspondent" writes: Absence from England has prevented me from seeing The Hospital, and consequently my sisters' answers to my observations upon the modern nurse. I think some of these said answers call for a rejoinder on my part, as they certainly are misleading, and do not at all represent my views. To start with my objections to outdoor uniforms. These are simply founded upon the conduct of some of the wearers. Aesthetically, I like uniforms; I admire the picturesqueness of a foreign street, with its sisters, its priests, its officers, its peasants, and its servants, all costumed in their respective fashions; and I wish we could imitate our French and German cousins therein. "A Wearer of the Red Uniform" finds that in my first article (August 19th, p. ccvii) I put a veto upon laughing, &c. "A nurse must not laugh, or when in omnibuses raise her voice so as to be heard by those sitting beside her; she must not, when in Regent Street or near the Burlington Arcade, be allowed to look in shop windows, and to think of going to a place of amusement would be far too great a crime." Where this red-garmented nurse found these views of mine I cannot say; I only hope her nursing is not as imaginative as her criticism, and that she does not read her directions as incorrectly as she has my article. Instead of protesting against nurses speaking in omnibuses for the benefit of their friends, my objections are "talking shop in a voice which everybody can hear"—talking, in fact, to the crowd. Accuracy being one of the most necessary qualifications of a nurse, let the red uniform critic re-read my first article, "Deterioration," and her letter objecting thereto; and then let her compare my charges with her answer. As to the "One Who Wears a Fringe," I fear she and I start from such totally different points of view that any harmony between us is impossible. She thinks "every woman's duty is to dress as prettily as possible and to look as charming as possible," and why should nurses be an exception to this rule? I see no reason whatever if it be a rule; but from my point of view I doubt this being the duty of women of any class or profession. It seems to me that "duty" lies in higher walks than those of "dressing prettily and looking charming." I quite agree in honouring all the noble women who are acting as nurses, but it is just these "noble women" who would be the first to repudiate the idea that they wear a uniform because it is becoming and charming; they, like us who wear worldly attire, wish to pass in the crowd unnoticed. It is the wearing of uniform which is noticeable, and the parading of the profession, which is objectionable. I have not the slightest objection to nurses going to theatres, but I have the strongest objection to any woman who goes to places of amusement alone, or with a woman friend, decking herself out in attractive garments of one form or another. Women who must be their own guardians should dress in such a way as to pass in the crowd.

### Minor Appointments.

Bristol Women and Children's Hospital.—Miss F. M. Johnson has been made Sister at this hospital. She was trained at the Royal Albert Edward Infirmary, Wigan, where she afterwards held the appointment of Sister. Miss Johnson has good testimonials.

### For Reading to the Sick.

### CLOUDS.

The many weeks and months of uninterrupted fine weather and cloudless skies that we have experienced this year, shows us by its effects that we may "have too much of a good thing," as the old saying has it. The parched earth is panting for rain and dull weather, while the dwellers upon it are fainting and discontented at the continual sunshine and drought. God in His good providence will doubtless soon send bountiful showers, and we shall be joyfully singing, "Thanks be to God for He loveth the thirsty land." In the meantime, however, the heavens will be black with clouds, and wind and tempests, which will bring desolation in their train. When we read in the 104th Psalm that "God watereth the hills from His chambers," we simply think of the effects; that by doing so "He causeth the grass to grow for the cattle, and herb for the service of man, that He may bring forth fruit out of the earth," while we forget the lowering clouds and darkness which are the forerunners of abundance of rain, and which give the whole world an aspect of gloom and sadness. The last thought which would occur to us would be, that the clouds are the chambers of God, that He dwelleth in thick darkness, and that those dark walls enclose a presence so bright and glorious that no man could see it and live. The clouds are called His "chambers" because God is said to abide there. We know that Jehovah the Omnipresent is not confined to any particular spot, but is present everywhere, and makes His presence felt by the blessings or corrections He sends, or allows to be sent upon His creatures. Sometimes the heavy clouds pour out destruction in grievous floods or scathing lightning, while again it is only a precious rain which they shed to refresh the earth when it is weary.

Our spiritual life is dealt with much in the same manner. We may be allowed to have great prosperity without sickness or misfortune of any kind. Year after year passes and nothing changes. We fall into no misfortune like other folk, neither are we plagued like other men. But is this state of things entirely for our good? Alas! no. We become dried up with selfishness; we lose sympathy with our suffering brethren; we think in our hearts that it is our own wisdom and goodness which keep us from sickness and distress; we bring forth no fruits to God's glory. But he has been only trying us to strengthen our faith and trust in Him, and when they fail and He hides His face from us in the clouds and thick darkness, we are in despair and cry out for the light. We have been so used to the bright shining of His countenance that when it is hidden from us we forget that the same glorious presence is waiting to soothe and cheer us. Let us remember that all is not gold that glitters, neither is all that is dark sorrowful. A house which on a winter's night looks dismal from without, is full of brightness and comfort within. "There is a silver lining to every cloud" is a beautiful idea, but not nearly so beautiful as the words of the Psalmist, "God watereth the hills from His chambers," for "we learn from them," as a modern writer says, "that every cloud is the abode of Deity, and it is not till it breaks that we see how full it is of mercy."

### Notes and Queries.

### Queries.

(203) *Great Toe.*—Kindly say who is the maker of instrument shown in The Hospital of September 9th, article headed "Treatment of Hammer Toe."—*Elderly Nurse.*

(204) *Birth.*—Please tell me where I can get a certificate of my birth?—*Ada.*

(205) *Training.*—Where can I get general training?—*Male Nurse.*

(206) *South Africa.*—Information as to nursing at the Cape wanted.—*N. C.*

### Answers.

(203) *Great Toe (Elderly Nurse).*—Mr. Schramm, 24, Great Castle Street, London.

(204) *Birth (Ada).*—Address your query to the registrar of births and deaths in your native place.

(205) *Training (Male Nurse).*—We regret that we know of no English hospital which trains men. It is a great pity that some progress is not made in this important direction.

(206) *South Africa (N. C.)*—Read article on "Nursing in South Africa" in The Hospital of September 16th.

### The Muses' Looking Glass.

### "DODO—A DETAIL OF THE DAY."

"An unknown quantity, which she could not gauge,"—such did love appear, from first to last, to the heroine of Mr. Benson's brilliant but terribly sad little sketch. And to us, too, as we ponder over this book, there comes much of the same sense of awe and of helpless ignorance which oppressed Dodo whenever she was brought face to face with this "unknown quantity." An unknown quantity it is not indeed—thank heaven for it!—to the generality of mankind; but who that reads this book—who, indeed, that looks at life, can fail to own that love cannot be "gauged,"—that the power to arouse and to keep it cannot be accounted for by the possession of those sterling qualities which we are taught to regard as "loveable?"

*Who, indeed, that looks at life.* We added this advisedly, for the supreme merit of this book is that it is real, that life —a certain side of life—as it is, and life as here presented to us, are one and the same thing. Dodo is of real flesh and blood; we feel, as we lay down the book, that we have seen and known her; and what is more remarkable, we fully believe in her power to fascinate, and to inspire deep and lasting love; we feel that in spite of her shallowness, her cold-bloodedness, her selfishness, she would break hearts and wreck lives, were she to appear among us, just as she did in the story. It is a wonderful sketch, this portrait of Dodo, with her beauty, her winning ways, her amazing vitality, her quick wit, and original vein of humour; and on the other hand, her want of natural affection (we cannot recall one person for whom she felt anything that deserved to be dignified by the name of love), her intense self-centredness, her almost inconceivable light-mindedness—a light-mindedness which could allow her, the very morning after she had invited her old lover to run away with her, and had witnessed his terrible conflict between love for her and duty towards her husband, to skip before her cheval-glass, admiring her figure and her new hat, and humming to herself, "Just look at that, just look at this; I'm really not so much a miss!"

When her baby comes, she only feels "a certain pleased sort of proprietorship in the little pink morsel," because it must be an important personage some day; and when it dies, her first feeling is one of passionate resentment for herself. "It was not fair; she had just been released from two tedious months of inactivity, only to be caught again. . . . She wanted to revenge herself on something." And after her first quarrel with her husband, her first frank avowal that his wishes were nothing to her—an avowal which showed him at last that she did not love him, and which entered like iron into his soul, the tragedy of it passed over her head; she saw nothing but the "unpleasantness" of being on bad terms with her husband, and having asserted her determination to go her own way, proceeded to make up the quarrel. "He was standing with his hands clasped behind him near the door. She laid her hands on his shoulder, and gave him a little shake. 'Now, Chesterford, I'm going to make up,' she said. . . . 'I hate quarrelling, and you don't look as if it agreed with you. Kiss me this moment. No, not on the top of my head. That's better. My carriage ought to be ready by this time, and you are coming with me as far as Prince's Gate.'"

What is there loveable about such a woman? And yet—and yet—she was loved, almost worshipped. Her women friends were genuinely attached to her—even the one who described her as "beautiful, unscrupulous, dramatic, warm hearted, cold-blooded, and a hundred other things." And as for her lovers, in Chesterford's eyes she could do no wrong; he was only filled with pity for her when he found that she "had got tired of him;" and on his death-bed he could call her his "darling," his "sweet own wife," and tell her that she "had been very good to him, and had given him more happiness than he ever thought he could have had." And Jack—Jack, who saw her faults, felt "an irritated disgust" for her now and again, and even came to wondering once whether she was a devil, a tiger, or a woman, finally deciding that at any rate "the tigress element was not wholly absent"—even he could never shake himself free from his attachment to her—unless, indeed, that last cruel blow which she dealt him in the concluding chapter may have finally disillusioned him. Perhaps it did, for Dodo grows as the book goes on—grows, not in grace, but in hardness of heart. No one, surely, could have loved her if she had been consistently and solely the selfish, heartless, ideal less being which her story proves her to have been as a whole. When Jack first loved her, he saw signs of something in her deeper and better than often appeared, and "though the shallows were commoner than the depths, and their presence was sometimes indicated bya rather harsh jarring of the keel, yet he believed fully and sincerely in the dark, mysterious depths for love to lose itself in."

Nor did he altogether deceive himself. The author speaks somewhere of an "undeveloped germ of simplicity" in Dodo; and he shows us clearly that there were many more of those undeveloped germs—a germ of religion; which made her feel, after going to a little country church on a summer evening, that she'd "never do anything naughty any more"; a dim, uneasy consciousness that there were higher things than her own selfish aims and objects, as when she burst into passionate tears on the evening of her engagement, and again when from the piano there crashed out "a great kingly motif, strong with the strength of a man who is pure and true," making her look across at Chesterford, in her eyes "the suggestion of unshed tears, and a look of questioning shame." Had she cultivated those germs, had she sought that "grace of God" which her friend, Mrs. Vivian, told her had wrought so great a change in herself, who can doubt that she might have conquered self, and brought peace and happiness, instead of misery and shame, to those who loved her? But she "would none of these things." When better thoughts came, she drove them away; when Jack urged her to look at their love as something sacred, and for a moment impressed and moved her, she ended off with, "I believe I have an ideal—which I never have had before—something to respect and to keep very clean. Fancy me with an ideal! Mother wouldn't know me again; there never was such a thing in the house." Her conscience, too, was patronised as being a great convenience, "quite good, without being at all priggish. It isn't exactly what you might call in holy orders, but it is an ecclesiastical layman, and has great sympathy with the church. A sort of lay reader, you know."

And thus, with her better nature crushed down, or only brought out to be laughed at, her worse nature encouraged, she made a tragedy of her own life, and of the lives of others; though she would have said, when first we knew her, "Is thy servant a dog that he should do this thing?" In spite of its brilliance, its cleverness, its humour, this book is a sermon—a solemn warning, whether the author meant it or not, to those who choose the evil and reject the good; and as such we hope it will be taken.

E.

### Wants and Workers

Will someone inform Nurse H. whether there is any school for massage or classes which a nurse could attend in Liverpool?

Can anybody tell Sister T. where a "Shakespeare" cot can be procured, also the probable cost?

*Infant Orphan Asylum, Wanstead.*—If any of our readers have not yet promised their votes for the next election at this institution, we can strongly recommend a very deserving case, that of Grace Lillian Lloyd, aged six years and two months. Her father, a clerk in the Supreme Court Pay Office, died after a few weeks' illness on September 5th last, leaving a widow and four children unprovided for. Mr. Lloyd was personally known to us as a clever, hardworking man, and but for his sudden and untimely end he would undoubtedly have made ample provision for his family. Proxies will be gladly received, and gratefully acknowledged, by the Editor.

### The Book World for Women and Nurses.

[We invite Correspondence, Criticism, Enquiries, and Notes on Books likely to interest Women and Nurses. Address, Editor, The Hospital (Nurses' Book World), 428, Strand, W.C.]

Much Cry and No Wool.

In the current number of the *Strand Magazine* is an "illustrated interview" with Luke Fildes, R.A. The interview is no worse than other "interviews," the victim is made to appear mildly egregious, but that of course is his own fault if he consent to be interviewed. The illustrations are not more misleading than usual. The slip of garden is made to appear a park, and the rooms are necessarily, but unkindly, deprived of what in an artist's house is their chief attraction, their colour scheme. What concerns us here is the engraving of Mr. Fildes' celebrated picture, "The Doctor," and Mr. Fildes' confessions regarding it. Mr. Tate wanted a very representative Fildes for his collection of modern English pictures. Mr. Fildes thought "The Doctor" a good subject. "He should be the actor in the little drama; father, mother, and child should only help to show him to better advantage." Then "I travelled to many places, from Devon to Inverness, to get thoroughly acquainted with the character of the cottages and the people. Whilst on my journeyings I had been picking up odds and ends of furniture—even the cup and basin were specially purchased. I made many sketches of fishers' huts, returned to town, and had the room built exactly to size at the far end of the studio. It was a most substantial structure—even the massive rafters were there—and I painted a great cloth to look like red bricks." Here we have the so-called Realistic school exhibiting its folly and proclaiming its own justification to be nonsense. Realistic pictures are "human documents" we are constantly told. Then why all this travel and travail, this trouble and expense to build up imitation cottages to show up one's sham doctor painted from a professional model, gazing with soul-stirring intensity at a very healthy child? At least hire a sick child—but no. They were Phyllis' curls, though the sleeping child was Mr. Fildes little Geoffrey. "When he wanted his morning sleep he used to be brought to the studio. As he slept I painted." Can anything be more beautifully calm than the morning sleep of a healthy child? Fancy making a fool of one's would-be hero, the doctor, by causing him to look with grave anxiety and watchful intensity at the normal morning nap of a child with round cheeks, unsunken eyes, and with its rosy palm unclosed! A child in pain and discomfort invariably clenches its fingers during sleep, and no doctor feels seriously anxious when its fingers are loose and straightened, as in the *un*realistic picture of "The Doctor."

SHORT NOTICES.

West Kirby as a Health and Pleasure Resort. (Willmer Brothers, Birkenhead.)

It is not certain that "Improvement Committees" are an unmixed boon to a sea-side village. West Kirby, it seems, is undergoing some of the processes connected with its transformation into a health resort; but it still retains the advantages of comparative quiet and decided economy, and though hardly needing a guide to its beauties may be recom-

**THE BOOK WORLD FOR WOMEN AND NURSES**—*continued.*

mended to any of our readers who still have their holiday before them.

Horne's Guide to Whitby. Illustrated. (Horne and Son, Whitby.)

This is a new and better edition of an excellent guide. The maps and illustrations leave nothing to be desired, and all matters of local information and interest are given with great accuracy and clearness of arrangement.

Bright Thoughts for Weary Hours. By Clara Bray. (Hodder Brothers.)

This inexpensive little volume will be found of use by many for reading to the sick, though it would have been more handy as a pocket volume. The meditations are simple and in good taste, and have the great merit of being short, and to the point.

Short Biographies for the People. Mrs. Hemans. (Religious Tract Society). Price 1d.

These little biographies, of which over a hundred have been already issued, are well put together, and clearly printed, presenting a wonderful pennyworth of attractive reading. They would be acceptable in many lonely rooms, and comprising a great variety of notable personages ranging between Polycarp of Smyrna and Rob Roy Macgregor, it should not be difficult for everyone to suit his own taste. This story of Mrs. Heman's life is told with simplicity and effect, and includes some of her best known poems.

The Zenana or Woman's Work in India. (Partridge and Co.) Price 1d. monthly.

This new and attractive little paper is designed to give information monthly about the Zenana Bible and Medical Mission. The thought of "the hundred and forty millions of women in India for whom (to quote Lord Roberts) skilled medical aid is at present an impossibility," should ensure a welcome for every effort to strengthen the hands of the workers among them, and "The Zenana" will no doubt soon be popular among a wide circle of readers.

The Problem of Human Suffering. By the Rev. T. Sterling Berry. (Religious Tract Society.)

A well thought out little pamphlet, dealing with what may be called the advantages of suffering. The style is hardly light enough to recommend it to the casual reader, but it will well repay rather deeper attention.

### AN INTERESTING CATALOGUE.

Anyone wishing to make their friends presents in the way of books should consult the little "Catalogue of Gift Books," published by Messrs. Cassell and Co. It contains a very complete list of works new and old, and is, moreover, embellished with specimen illustrations. We are constantly welcoming from Messrs. Cassell's hands old friends in new and charming dress, and we find this to be the case again in glancing down the present list, also meeting with many and very interesting new publications. The small booklet is sufficiently attractive-looking with its bright red cover.

